# Single-cell sequencing reveals the potential oncogenic expression atlas of human iPSC-derived cardiomyocytes

**DOI:** 10.1242/bio.053348

**Published:** 2021-02-15

**Authors:** Minglin Ou, Min Zhao, Chunhong Li, Donge Tang, Yong Xu, Weier Dai, Weiguo Sui, Yue Zhang, Zhen Xiang, Chune Mo, Hua Lin, Yong Dai

**Affiliations:** 1 Central Laboratory, Guangxi Health Commission Key Laboratory of Glucose and Lipid Metabolism Disorders, The Second Affiliated Hospital of Guilin Medical University, Guilin 541000, China; 2 Clinical Medical Research Center, The Second Clinical Medical College, Jinan University, Shenzhen People's Hospital, Shenzhen 518020, China; 3 GeneCology Research Centre/Seaweed Research Group, School of Science and Engineering, University of the Sunshine Coast, Queensland 4556, Australia; 4 Guangxi Key laboratory of Metabolic Diseases Research, Central Laboratory of Guilin No. 181 Hospital, Guilin 541002, China; 5 College of Natural Science, University of Texas at Austin, Austin 78712, Texas, USA; 6 College of Life Science, Guangxi Normal University, Guilin 541006, China

**Keywords:** Single-cell transcriptomics, Oncogene, Tumor suppressor gene, MYC, TP53

## Abstract

Human induced pluripotent stem cells (iPSCs) are important source for regenerative medicine. However, the links between pluripotency and oncogenic transformation raise safety issues. To understand the characteristics of iPSC-derived cells at single-cell resolution, we directly reprogrammed two human iPSC lines into cardiomyocytes and collected cells from four time points during cardiac differentiation for single-cell sequencing. We captured 32,365 cells and identified five molecularly distinct clusters that aligned well with our reconstructed differentiation trajectory. We discovered a set of dynamic expression events related to the upregulation of oncogenes and the decreasing expression of tumor suppressor genes during cardiac differentiation, which were similar to the gain-of-function and loss-of-function patterns during oncogenesis. In practice, we characterized the dynamic expression of the *TP53* and Yamanaka factor genes (*OCT4*, *SOX2*, *KLF4* and *MYC*), which were widely used for human iPSCs lines generation; and revealed the co-occurrence of *MYC* overexpression and *TP53* silencing in some of human iPSC-derived *TNNT2*+ cardiomyocytes. In summary, our oncogenic expression atlas is valuable for human iPSCs application and the single-cell resolution highlights the clues potentially associated with the carcinogenic risk of human iPSC-derived cells.

## Abstract (precis)

**Summary:** The single-cell expression atlas in the cardiomyocytes generated from human iPSCs provide potential carcinogenic information for the clinical application of human iPSC-derived cells.

## INTRODUCTION

In general, the human induced pluripotent stem cells (iPSCs) generated from individual somatic cells have similar features to the features of embryonic stem cells (Féraud et al., 2016). Therefore, these iPSCs can be differentiated into multiple human cells with the benefit of circumventing the immune rejection barrier, which may be a key source of cell-based therapies (Xu et al., 2009). In principle, cardiomyogenic lineages generated from iPSCs could improve heart function, and allogeneic iPSC-derived cardiomyocytes transplantation is effective to regenerate the infarcted heart in nonhuman primates and humans (Chen et al., 2016; Miyagawa and Sawa, 2018; Shiba et al., 2016; Tohyama and Fukuda, 2017b). At the molecular level, mesoderm inductions are controlled by multiple signaling pathways including activin-Nodal, BMP, Wnt and FGF, which will be subsequently induce cardiac specification by inhibiting the Wnt8 and TGFβ3 pathways (Burridge et al., 2014; Lan et al., 2013). Based on these principles, a number of effective methods were used to produce pure troponin T-positive (*TNNT2*+) cardiomyocytes. However, numerous technical and scientific challenges remain before human iPSC-derived cardiac cells can be used for clinical therapy (de Lázaro et al., 2014). One of the most important roadblocks is the potential tumorigenicity, in which the cells will reprogram and will have a high rate of tumor formation in those iPSC-derived cells in animal models (Ito et al., 2019; Liu et al., 2013; Martins et al., 2014; Nori et al., 2011).

In recent years, contamination of undifferentiated iPSCs has been identified as an important cause of tumor formation in iPSC-derived cell-based therapies, but that is not the whole story (Tohyama and Fukuda, 2017a). In practice, human iPSCs were often generated from somatic cells by the viral-based delivery of Yamanaka factors (Oct4, Sox2, Klf4, and c-Myc), which are pioneer transcription factors involved in cell differentiation (Kim et al., 2015; Kuzmich et al., 2015; Liu et al., 2013; Riggs et al., 2013). The pioneer transcription factors have also been confirmed to take an active part in genomic instability. Additionally, alteration in gene expression caused by genomic instability play key roles in the tumorigenicity of human iPSC-derived cells (Iida et al., 2017). However, all of these findings were obtained from population cell analyses at the tissue or organ level, and a genome wide survey of human iPSC-derived cells at a single-cell resolution is still lacking. Furthermore, these events may occur only in a portion of cells during cell differentiation and it is difficult to understand their characteristics by population cell-based analysis (Seitz et al., 2011; Trumpp, 2006). Recently, many studies have indicated that single-cell sequencing is a powerful analytical tool to reveal previously unknown molecular events, including *HOPX* was found playing important roles in the maturation of iPSC-derived cardiomyocytes; this provides the possibility for us to understand the oncogenic expression in human iPSC-derived cells at the single-cell level (Alvarez et al., 2012; Liu et al., 2017b; Wang and Navin, 2015; Ziegenhain et al., 2017; Friedman et al., 2018). However, limited cancer genes information on human iPSC differentiating towards cardiomyocytes at single-cell level have been reported in the literature.

In this study, we directly reprogrammed human iPSC lines into cardiomyocytes and analyzed their transcriptional profiles at a single-cell resolution. By focusing on those key oncogenes and tumor suppressors, we performed unbiased cell clustering, and trajectory analyses to explore the key oncogenic events. This oncogenic expression atlas in the cardiomyocytes generated from human iPSCs provide novel information for the clinical application of human iPSC-derived cells at a single-cell resolution.

## RESULTS

### Single-cell analysis of marker gene expression signatures in the cardiac differentiation from human iPSCs

To explore the iPSC reprogramming at the single cell level, we performed a systematic cell isolation and sequencing. In total, we obtained 2,066,741,896 high-quality clean reads from 32,365 cells in the four groups of samples, including 495,716,337 reads from 11,281 cells, 510,774,356 reads from 6,466 cells, 505,141,340 reads from 8650 cells, 555,109,863 reads from 5968 cells collected on day 0, day 2, day 4 and day 10 of cardiac differentiation, respectively. More than 90% of these short reads were confidently mapped to the human genome and generated an average read depth per cell of 63,857. On average, 3751 genes and 16,217 UMI were detected per cell. Regarding the sequencing data in the differentiation time points, we detected 23,686, 24,026, 23,982 and 23,958 genes in the sample groups collected from day 0, day 2, day 4 and day 10, respectively. Overall, we detected 26,554 unique genes after quality control, which were differentially expressing in the cells of different time points (Fig. S1).

**Fig. 1.**
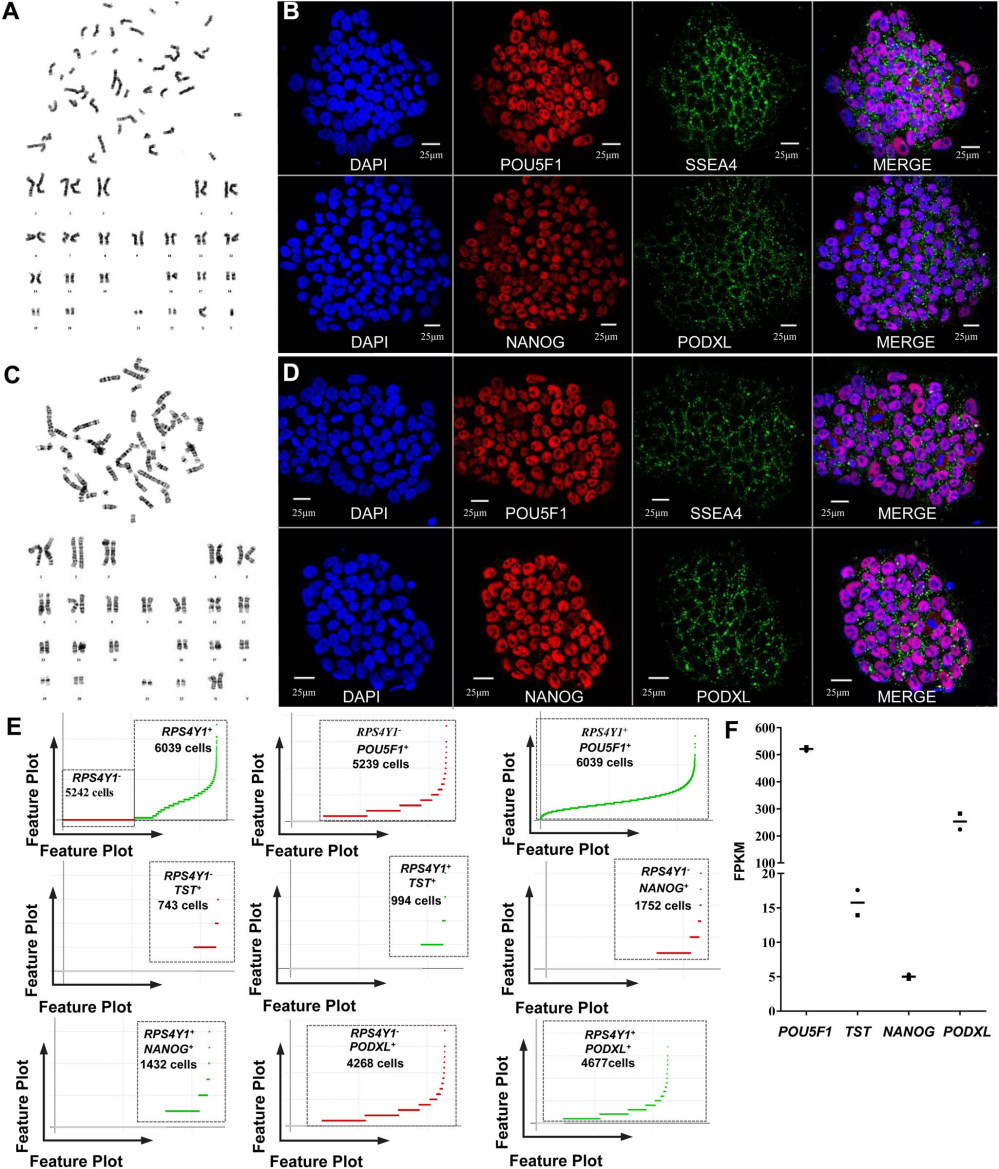
**The general characteristics of the two human iPSC lines.** (A) The cell line 1 carried a normal male karyotype; (B) immunofluorescence analysis confirmed that the common pluripotency markers positively expressed in cell line 1. (C) The cell line 2 carried a normal female karyotype. (D) immunofluorescence analysis confirmed that the common pluripotency markers positively expressed in cell line 2. (E) Feature plots of cell barcodes of *RPS4Y1* and pluripotency marker genes on day 0 of cardiac differentiation, the scale on the Y-axis was linear expression. (F) Bulk RNA sequencing confirmed the expression of pluripotency marker genes in the two cell lines. The X-axis represented the genes and Y-axis represented the expression level of genes; the square represents cell line 1, the dot represents cell line 2, and the short line represents the mean FPKM of the two cell lines.

**Fig. 2.**
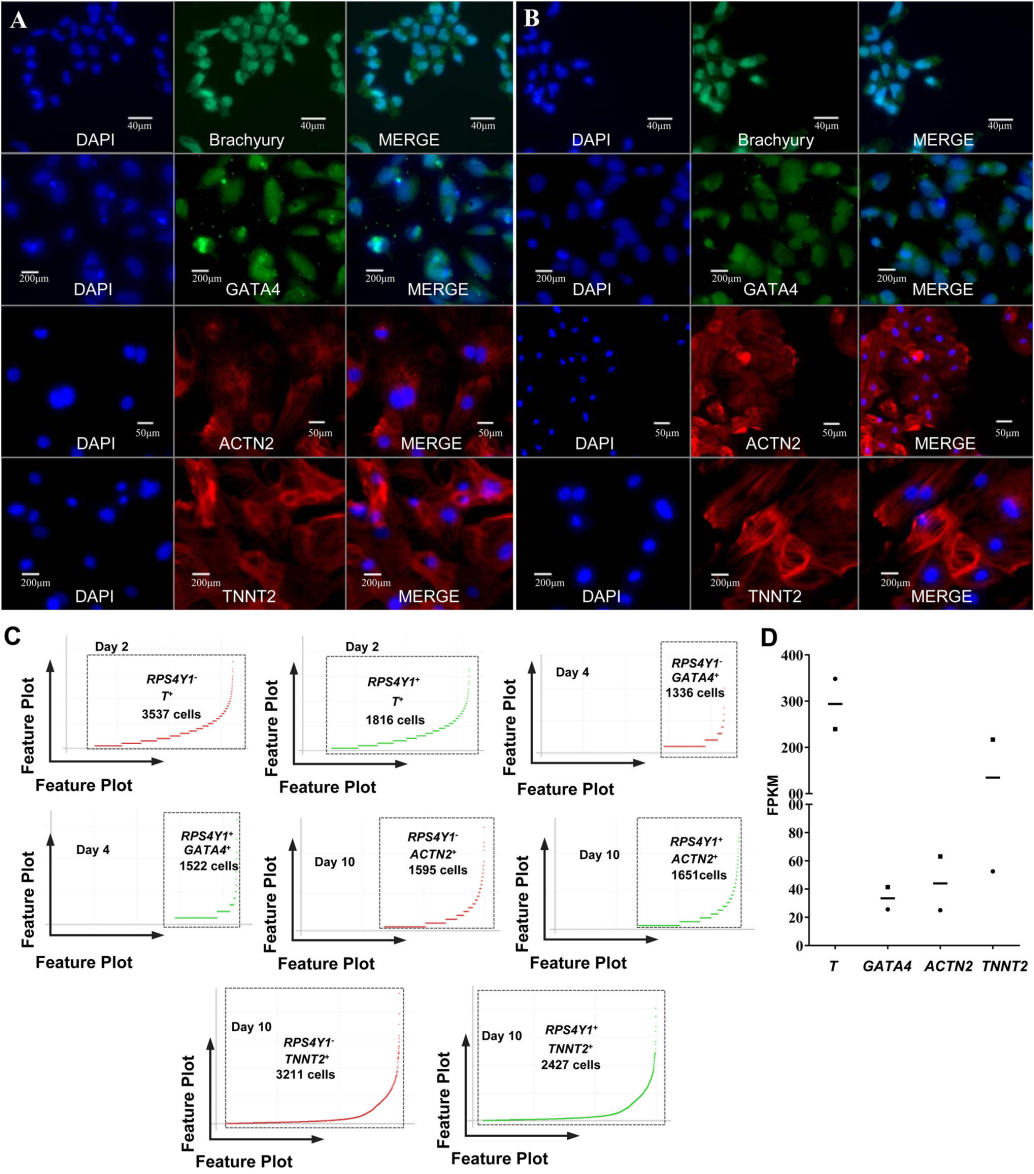
**Marker gene expression at the four time points of cardiac differentiation.** (A) Cell line 1 and (B) cell line 2 confirmed by immunofluorescence analysis showing mesodermal markers Brachyury expression on day 2, cardiac precursor marker GATA4 expression on day 4, and cardiomyocyte marker ACTN2 and TNNT2 expression on day 10 in the cardiac differentiation of the two human cell lines. (C) Feature plots of cell barcodes of *RPS4Y1* and marker genes in the cardiac differentiation on day 2, 4 and 10 (the Y-axis was linear scale). (D) Bulk RNA sequencing confirmed the expression of marker genes at different time points of the cardiac differentiation. The X-axis represented the genes and Y-axis represented the expression level of genes; the square represents cell line 1, the dot represents cell line 2, and the short line represents the mean FPKM of the two cell lines.

The two human iPSC lines were typical iPSC colonies, verified carrying a normal male karyotype and a normal female karyotype (Fig. 1A,C; Fig. S2). To confirm known marker gene expression signatures, human iPSCs and their differentiated cultures were analyzed by single-cell sequencing and immunofluorescence (Burridge et al., 2012). Here, the gene-cell barcode matrix of each single sample group was used for PCA and tSNE analysis. Considering the bulk RNA sequencing data and the data from the literature, the Y chromosome gene *RPS4Y1* was chosen to be used to distinguish the two human iPSC lines, such as the *RPS4Y1*-positive expressed cells were registered as generation from the cell line 1 and the cells without *RPS4Y1* expression were registered as generation from the cell line 2 (Dierselhuis et al., 2010; van den Berge and Sijen, 2017). Our immunofluorescence and single-cell sequencing data confirmed that the two human iPSC lines were undifferentiated and had a positive expression of the general pluripotency markers including *POU5F1* (also known as OCT4), *SSEA4*, *NANOG*, and *PODXL* (also known as *TRA-1-60*) (Fig. 1B,D,E). In cardiac differentiation, we found that the mesodermal markers [*T* (Brachyury), Fig. 2A–D] and the cardiac precursor marker (*GATA4*, Fig. 2A–D) were positively expressed on day 2, and day 4, respectively. Furthermore, we successfully generated cardiac α-Actinin-positive (*ACTN2*+) and TNNT2-positive (*TNNT2*+) cardiomyocytes on the day 10 (Fig. 2A–D). The expression patterns of these critical marker genes, including *POU5F1*, *TST*, *NANOG*, *PODXL*, *T*, *GATA4*, *ACTN2* and *TNNT2*, were also confirmed by bulk RNA sequencing (Figs 1F and 2C). In other words, our single-cell sequencing was sensitive enough to detect the known marker genes in our experiments where cardiac differentiation was reproduced. These results indicated that our cardiac differentiation was successful and that human iPSC-derived cardiac cells may come from mesoderm differentiation and cardiac specification, which is in agreement with the previously known literature (Burridge et al., 2012).

### Identification of cell subpopulations and the reconstruction of cardiac differentiation trajectory in a pseudo-time manner

**Fig. 3.**
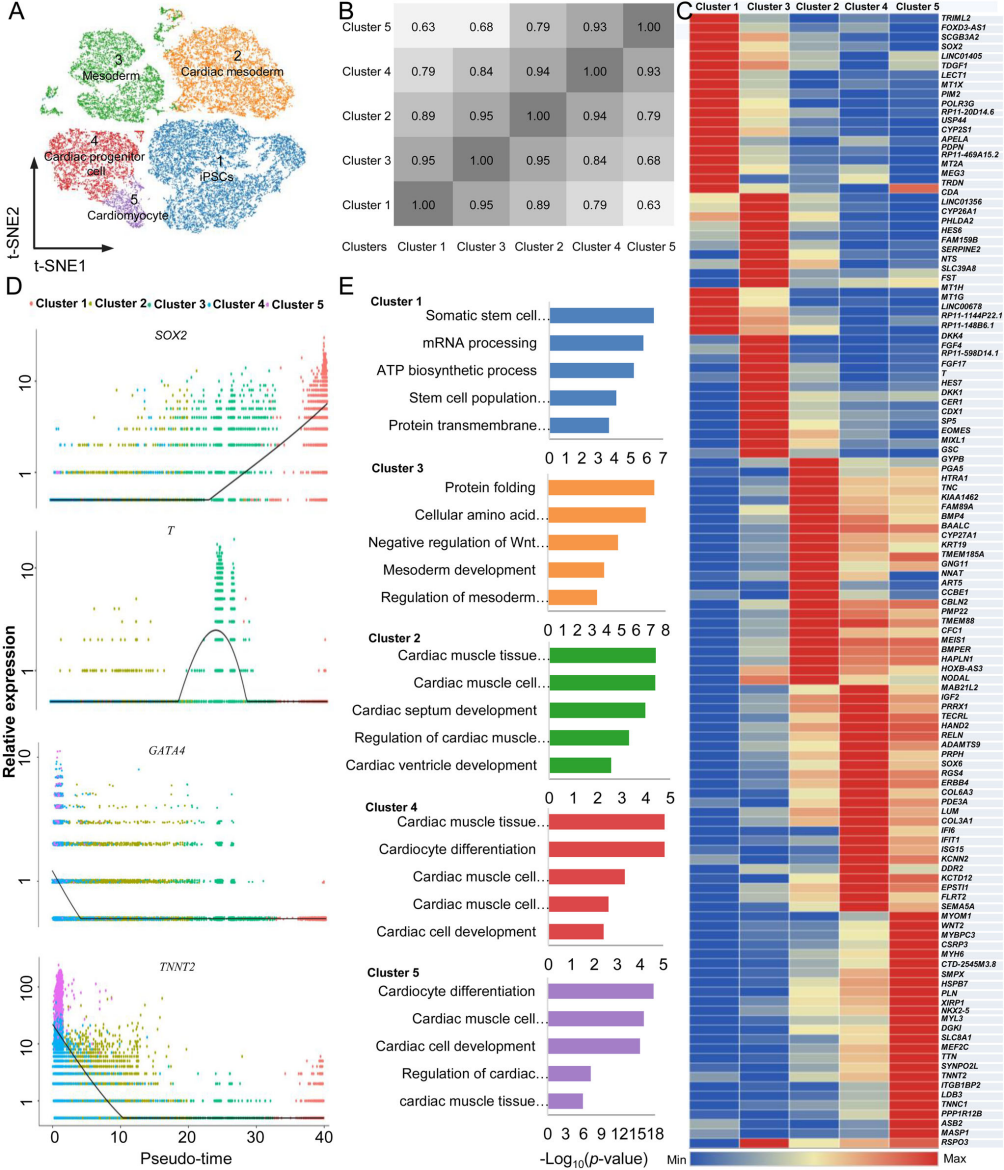
**The cell clusters and reconstruction of the differentiation trajectory of the whole cardiac differentiation from human iPSCs.** (A) t-SNE displaying the identified five clusters in the whole cardiac differentiation. (B) The correlation between the identified cell clusters. (C) The density plots of marker gene expressions in the five identified subpopulations in a pseudo-time manner. (D) A heatmap displays the cluster-specific genes in the five identified subpopulations. (E) Gene ontology analyses reveals the enriched terms of each cell cluster.

To explore the cell subpopulations, we used Cell Ranger to normalize the UMI data to account for differences in sequencing depth across the four group of samples, and normalize each cell to the median. And then the pooled four groups of single cell sequencing data were visualized by Loupe Cell Browser. As shown in Fig. 3A, there were five transcriptionally heterogeneous clusters of cells expressing different stage-specific genes emerged in the cardiac differentiation from human iPSCs (Fig. S3). To understand the distance between the cell subpopulations identified by unsupervised clustering, we used Pearson correlation analysis of transcriptional profiles at a single level to compare the correlations in Cluster 1, Cluster 2, Cluster 3, Cluster 4 and Cluster 5. These distance measures indicated that the cells in Cluster 1 were similar to those in Cluster 3 (1.00 being identical); the cells in Cluster 3 were much more similar to the cells in Clusters 2, 4 and 5 than the similarity of the cells in Cluster 1 was to the cells of Clusters 2, 4 and 5 (Fig. 3B,C).

**Fig. 4.**
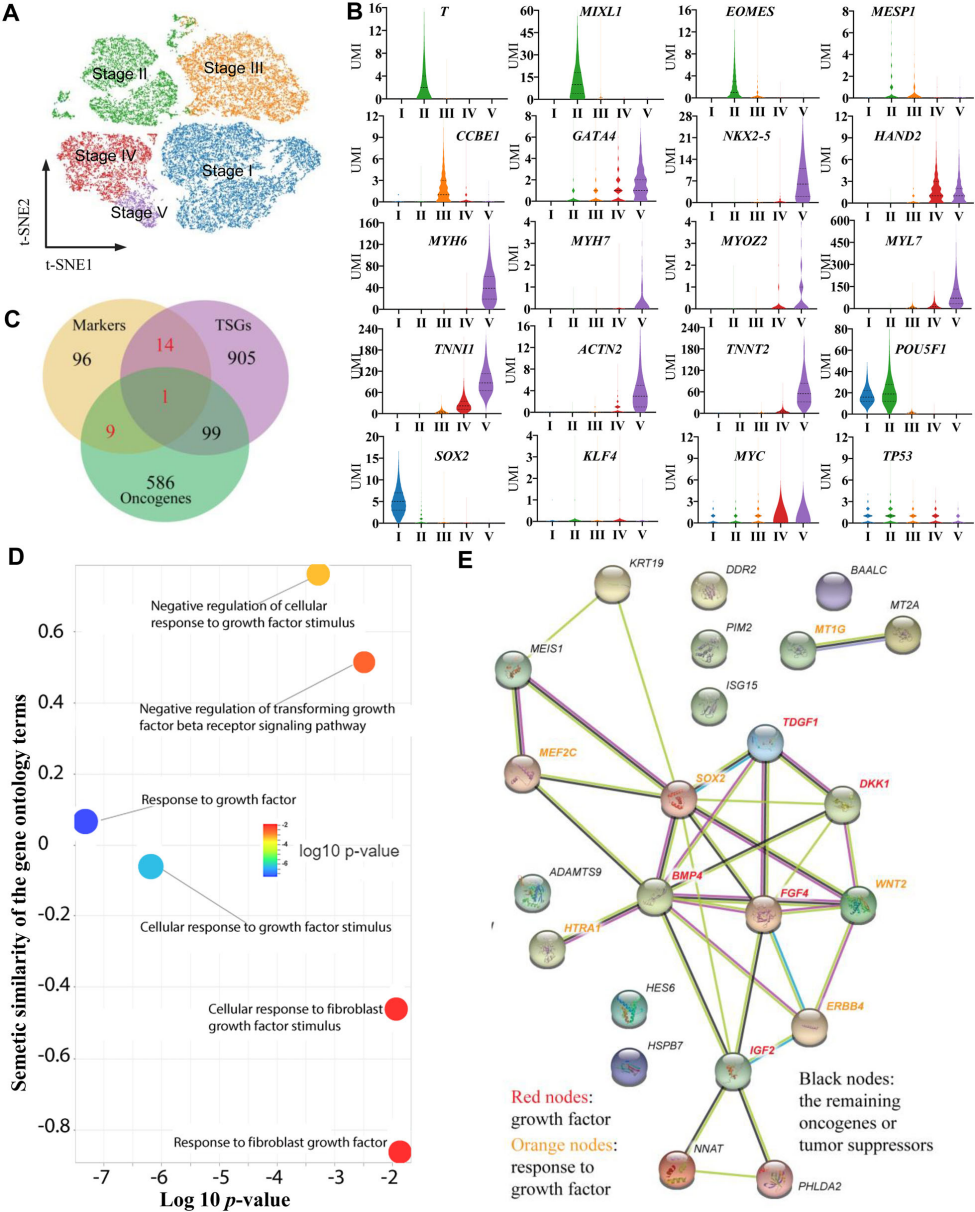
**The marker genes and their function in the five stages of cardiac differentiation.** (A) t-SNE projection displaying the identified five stages of cells. (B) A violin plot displaying the marker genes, the Yamanaka factor genes and *TP53* expression in the five stages. (C) The overlapping oncogenes and tumor suppressors with maker genes identified in this study. (D) The molecular interaction network for the 24 key oncogenic and tumor suppressive marker genes based on STRING interaction database. The light green color means the gene-gene interaction based on text mining results; the black color is based on the co-expression pairs; the protein homology-based interactions were indicated in blue color. The experimentally determined gene–gene interactions were marked with pink color. (E) The overrepresented gene ontology terms related to growth factor of the 24 key oncogenic and tumor suppressive marker genes.

In general, the profiles of gene expression in each cell might provide a molecular basis to explore the developmental trajectory in cardiac differentiation. Therefore, in addition to the distance analysis of different cell clusters, we tried to order the detected cells in pseudo-time manner. We found that each of the five cell clusters appeared in a particular order during reprogramming. However, our data also indicate that the different cell clusters did not appear after one another, and some cells in different clusters appeared at the same time, which indicated that the cells differentiation was continuous and asynchronous. Considering the distance between the clusters and the cardiac differentiation trajectory reconstructed by the pseudo-time manner, we defined the five clusters of cells in this cardiac differentiation from human iPSCs as five stages, including stage I, stage II, stage III, stage IV and stage V (Fig. 4A). In the stage I, there were 11,335 cells, 99.52% (11,281 cells) of which were from day 0, 0.26% (29 cells) were from day 2, 0.20% (23 cells) were from day 4 and 0.02% (2 cells) were from day 10; in the stage II, there were 7041 cells, 91.42% (6437 cells) of which were from day 2, 8.25% (581 cells) were from day 4 and 0.33% (23 cells) were from day 10; in the stage III, there were 8127 cells, 98.93% (8040 cells) of which were from day 4 and 1.07% (87 cells) were from day 10; in the stage IV, there were 4675 cells, 99.87% (4669 cells) of which were from day 10 and 0.13% (6 cells) were from day 4; in the stage V, there were 1187 cells, 100.00% of which were from day 10. Although some cells from different days mixed in the same clusters, the majority of cells in the same clusters were from the same sample collection time. These data indicated that asynchrony of cell division could be found in the cardiac differentiation and the stages defining by gene expression information in the clustering were consistent with actual cell differentiation time points in the cardiac differentiation from human iPSCs.

### Stage-specific gene expression signatures and their biology functions in cardiac differentiation from human iPSCs

The cardiac differentiation trajectory revealed the profiles of expected temporal-specific transcriptional characteristics (Fig. 3D). Based on these mapped five stages, we identified 120 stage-specific marker genes (Fig. 3D; Table S1). In fact, many of these marker genes were previously described as genes of lineage determination (Burridge et al., 2012). For example, in stage I, the human iPSC lines were positive for the expression of pluripotent markers; in stage II, previously reported markers, such as mesoderm genes *T*, *MIXL1* and *EOMES*, were successively activated, while expression of the pluripotent marker *SOX2* was gradually downregulated (Fig. 4B). However, the pluripotent gene *POU5F1* was still expressed at a high level during the induction from iPSCs to mesoderm cells (Fig. 4B). For development toward the cardiac mesoderm, we found that *MESP1* was mildly upregulated in stage II and stage III, and *CCBE1* was expressing in stage III and stage IV (Fig. 4B). The cardiac progenitor marker *GATA4* was found at early stage II, *NKX2-5* was expressed at the stage III, and they were continually expressed and upregulated at later stages (Fig. 4B). Moreover, *HAND2* was significantly upregulated in stage IV, which has been shown to play roles in promoting progenitor proliferation. Cardiac markers, such as *MYH6*, were found as early as stage III and were significantly upregulated in the last stages. Furthermore, we also found some cardiomyocyte marker genes, including *MYOM1*, *MYH7*, *MYOZ2*, *TNN*, *ACTN2*, and *TNNT2* (Figs 3C and 4B). These cell specific marker genes expressing in different cell subpopulations indicated that cardiomyocytes could be found on day 10 during cardiac differentiation.

To explore the gene expression dynamics at the single cell level during cardiac differentiation, we further focused on the genes that were significantly differentially expressed in different stages (Table S1). In fact, these genes may allow us to gain insight into a category of gene markers for different differentiation stages. For example, we found that cluster-specific genes of stage I were significantly enriched in 269 GO terms (*P*<0.05). Specifically, those genes were gradually downregulated from the stage I and involved in biological regulation, such as ‘stem cell population maintenance’ (i.e. *SOX2* and *TDGF1*) (Fig. 3E). Subsequently, the GO terms of ‘cellular amino acid metabolic process’ and ‘protein folding’ were enriched in stage II, and the genes that were transiently upregulated in this stage and finally downregulated in the later stages were enriched in ‘mesoderm development’ (i.e. *T*, *HES7*, *DKK1*, *DKK1*, *MIXL1*, and *EOMES*) and ‘negative regulation of Wnt signaling pathway’ (i.e. *GSC*, *DKK1*, and *DKK4*) (Fig. 3E). In stages III and IV, there were 467 and 549 significantly enriched GO terms, respectively, and the cluster-specific upregulated genes (i.e. *BMP4*, *TNNC1*, *SOX6* and *ERBB4*) were enriched in ‘cardiac muscle tissue development’. Finally, *MEF2C*, *MYH6*, *NKX2-5*, *TTN*, *TNNT2*, and *PLN* were activated in stage III or IV, and these upregulated genes were continually expressed at the highest level in stage V (Fig. 3E). Interestingly, these genes were enriched in cardiac development-related GO terms, such as ‘cardiac muscle tissue development’ and ‘cardiomyocyte differentiation’ (Fig. 3E). These data revealed that the cardiomyocytes may begin with the generation of mesoderm and develop through mesoderm speciation and cardiac specification, and well-known markers of cardiomyocytes can be found in stage V (Burridge et al., 2012).

### Potential roles of the stage-specific marker genes related to oncogenic transformation

To explore the potential roles of the stage-specific marker genes related to oncogenic transformation, we checked the oncogenic and tumor suppressive role of those identified marker genes by overlapping them to the oncogenes and TSGs gene list from the databases of (ONGene and TSGene 2.0) (Liu et al., 2017a; Zhao et al., 2016). As shown in Fig. 4C, we found a total of 24 marker genes with oncogenic or tumor suppressive roles. Further gene molecular network (Fig. 4D) and gene ontology enrichment analysis (Fig. 4E) revealed that these 24 genes were enriched in growth factor-related pathways, For example, *FGF4*, *BMP4*, *DKK1* and *TDGF1* present in our study were also identified as promoters of tumor growth (Katoh, 2007; Selga i Coma et al., 2009). These genes are clustered in the central of the molecular network to initial the potential regulatory events. In addition, ‘response to growth factor’ and ‘cellular response to growth factor stimulus’ were identified to have the lowest *P*-values. Basically, there are some cellular changes during the cell differentiation process as a result of a growth factor stimulus. For example, cardiac marker gene expression is strictly driven by growth factors, and the products of our enriched pathway genes, *FGF4* and *BMP4* are well characterized growth factors regulating cardiogenic precursor differentiation (Cai et al., 2003; Zwi et al., 2009). Growth factor-related pathways are also well-known regulators of various cancer processes. These results indicated that tumorigenicity-related pathways can be found in our study.

**Fig. 5.**
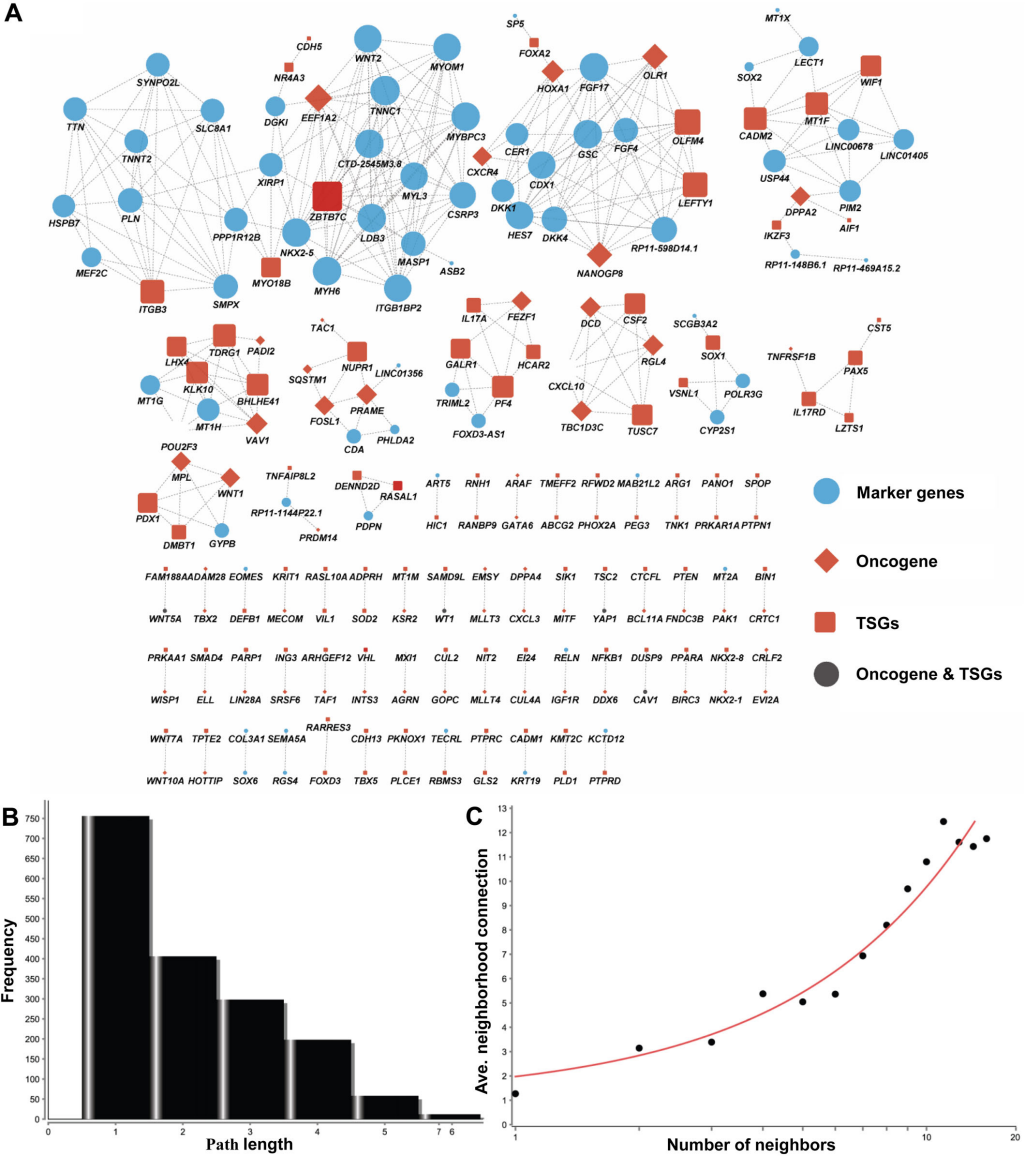
**Co-expression network for all the oncogenes, tumor suppressor genes and marker genes.** (A) The node size represents the number of connections and is larger when the number of connections is greater. The network represents the molecular function-based relationship between these marker genes and those important cancer genes in cardiac cell differentiation. (B) Distribution of the shortest path length. (C) The number neighbors distribution of the nodes in the network.

For linking our marker genes to those potential oncogenic genes, we built a co-expression network between all of the identified 120 marker genes and all of the oncogenes and tumor suppressors. By checking their expression among all the cells, we defined those genes with a similar expression pattern as the co-expressing genes. In detail, we combined all the marker genes, oncogenes and tumor suppressive genes and extracted their average expression values (UMI). In total, we had a total 1734 genes for further Spearman's correlation analysis based on the expression pattern in the five consecutive stages (clusters). For all the resulting *P*-values, we further checked the FDR to correct the statistical significance of multiple testing. From a total of 3,006,756 expression pairs, only 378 coexpression pairs satisfied our criteria that the expression correlation scores were greater than 0.99 and the FDR adjusted *P*-values were less than 0.01. In total, we identified 219 genes (nodes) in our network (Fig. 5A), including 69 marker genes. Because many pairs are not connected to each other, the majority path length is 1 or 2 steps (Fig. 5B). We also found that the number of neighbors in the network was corrected well with the averaged co-expression pairs in the network (Fig. 5C). This network not only presented the global view for the 69 makers with cancer gene roles, but also identified a number of functional modules. For example, there were two modules centered by the grow factors *FGF4* and *WNT2*. In our present study, *FGF4* and *WNT2* had temporal-specific transcription during stage II and stage V, respectively, and they were obviously some important target regulators in our cardiac differentiation experiments (Meilhac et al., 2014). These results indicated that there are some similar characteristics between carcinogenesis and cardiac differentiation from human iPSCs.

### Dynamic expression of oncogenes in the cardiac differentiation from human iPSCs

**Fig. 6.**
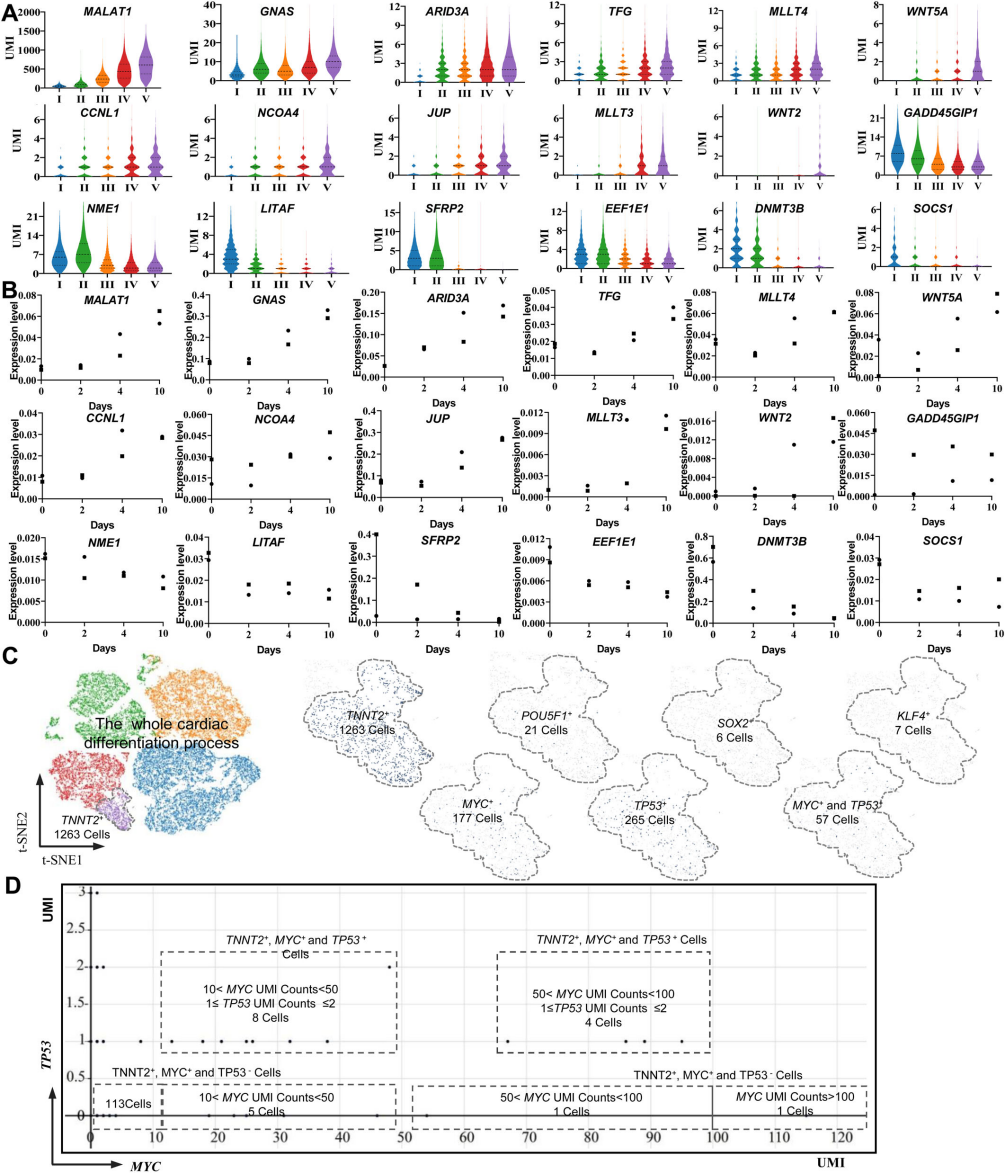
**Dynamic expression of oncogenes and TSGs in the cardiac differentiation from human iPSCs.** (A) A violin plot displaying the continuously upregulated expression of oncogenes, and the downregulated expression of TSGs detected by single-cell sequencing. (B) The displayed genes at different time points by the violin plot were confirmed by bulk RNA sequencing. The X-axis represented the cardiac differentiation time and Y-axis represented the relative expression level of the genes comparing to *GAPDH*, the square represents cell line 1, the dot represents cell line 2. (C) t-SNE was used to identify *TNNT2*+ cardiomyocytes with Yamanaka factor gene or *TP53*-positive expression in the last stage of cardiac differentiation. (D) A feature plot of the *MYC* and *TP53* expression signatures displaying the *TP53* expression status in the *MYC*+ cardiomyocytes (*TNNT2*+); the horizontal coordinates represent the expression level (UMI) of *MYC*, the vertical coordinates represent the expression level (UMI) of *TP53*, and the dots represent the combinatorial expression patterns of *MYC* and *TP53*.

To understand the dynamically oncogenic expression signatures in the cardiac differentiation trajectory, we overlapped the genes, which were detected in different stages, to the databases of ONGene (Liu et al., 2017a). We found 695 oncogenes that were expressed at different stages of our study. The majority of oncogenes [(695–189)/695=72%] were less than 1.00 UMI/cell in cardiac differentiation. There were 189 oncogenes (189/695=18%) with expression level of UMI/cell >1.00 were found in the five differentiation stages, 15 of which reached an average UMI/cell >10.00 in stage V (Table S2). Previous studies have revealed that the key regulators in cell development and carcinogenesis are often oncogenes that continuously increased their expression levels during cardiac differentiation (Yang et al., 2009). Therefore, we are further focused on oncogenes with continuous upregulation patterns among the 189 genes (Fig. 6A,B). In the most expressed oncogenes, we found that *MALAT1* was expressed at the highest level (UMI/cell=576.07) (Fig. 6A,B), and it was continuously upregulated from the beginning of cardiac differentiation (*P*<0.001). *MALAT1* encoded a bona fide long noncoding RNA and was previously identified as a prognostic marker for cancer metastasis (Gutschner et al., 2013). In addition, *MALAT1* might be regulated by *MAPK1* via the PI3K/AKT signaling pathway and might promote cardiomyocyte proliferation (Zhao et al., 2015). Interestingly, although *MAPK1* was expressed at different stages in this study, it was expressed at a very low level (UMI/cell <0.61). Therefore, there might be some novel upstream regulators for *MALAT1* or some intermediate regulatory mechanism to refine the relationship between *MAPK1*, *MALAT1* and cardiac generation. However, there were 13 oncogenes that continuously decreased their expression level during the cardiac differentiation, and the roles of dynamic expressed oncogenes needed to be further studied.

More interestingly, we also detected the Yamanaka factor genes, which were used for the generation of our two human iPSC lines expressing a limited amount of generated *TNNT2*+ cardiomyocytes in stage V (Fig. 6C). Their expression levels were relatively low compared to that of *MALAT1* (*P*<0.001), as their average UMI counts in the whole captured cells were 0.02 UMI/cell (*POU5F1*), 0.05 UMI/cell (*SOX2*), 0.01 UMI/cell (*KLF4*) and 0.85 UMI/cell (*MYC*) (Fig. 4B); the relative expression level of the Yamanaka factor genes was confirmed by bulk RNA sequencing. This finding indicated that the character of Yamanaka factor gene expression at the population cell level may be very difficult to accurately understand. However, at each single-cell level, single-cell sequencing identified 177 cells (14.9%) as *MYC*+, which highlights that tool is a powerful enough to detect oncogene expression signatures in both dominant and non-dominant cells. These data indicate that oncogenes were dynamically expressed throughout cardiac differentiation and that their roles involved in our cardiac differentiation are worthy of further identification.

### Dynamic expression of tumor suppressor genes (TSGs) in cardiac differentiation from human iPSCs

To understand the dynamic TSGs expression signatures in the cardiac differentiation trajectory, we overlapped the genes, which were detected in different stages, to the databases of TSGene 2.0 (Zhao et al., 2016). We found that 1020 TSGs were expressing in different stages of cardiac differentiation from human iPSCs, of which, 227 TSGs had expression levels of UMI/cell ≥1.00 at one or more stages. Then, we focused on those TSGs that continuously decreased their expression level during the cardiac differentiation because the downregulation or dysfunction of TSGs has been associated with cancer development (Chen et al., 2017; Klacz et al., 2016; Okugawa et al., 2013). In total, we found seven TSGs with continuously decreasing their expression levels by single-cell sequencing, such as *SFRP2* was expressed at high level in the early stage and at very low level in the late stage of differentiation (*P*<0.001), and it was confirmed by bulk RNA sequencing (Fig. 6A,B). *SFRP2* has been found to be a modulator in the Wnt pathway, and the inhibition of WNT signaling is necessary for cardiac differentiation (Kadari et al., 2015). The continuous downregulation of *SFRP2* may play a critical role in cardiac differentiation. However, *SFRP2* expression was associated with tumor formation, and the downregulated expression of this gene may increase the risk of malignancies (Kongkham et al., 2010). *DNMT3B* was also a typical gene that continuously decreased its expression level. Its product is DNA-methyltransferase-3B, which is identified association with the generation of DNA methylation in normal cell development and cancer development (Lee et al., 2005). The continuous downregulation of *DNMT3B* in our study may indicate that demethylation controlled by this gene may play important roles in the cardiac differentiation from human iPSCs. In addition, there were also 25 TSGs that continuously increased their expression levels their expression level during the cardiac differentiation, and the dynamic gene expression patterns may be difficult to broadly reflect the potential risk of oncogenesis. Then we analyzed the cell-to-cell heterogeneity of some well-known genes, such as TSG *TP53* and *MYC*. The expression of TSG *TP53* was UMI/cell <1.00 in the whole process of cardiac differentiation at cell population level, and *TP53* silencing happened in some human iPSC-derived cardiomyocytes (Figs 4B and 6D). By focusing on the 177 *TNNT2*+ and *MYC*+ cardiomyocytes, we found 53 cells with *TP53* expression (Fig. 6B,D). Our data indicate that the dynamic expression of those key TSGs was present in different single cells in cardiac differentiation, and oncogene activation and the dysfunction of suppressor genes may be important molecular events in human iPSC-derived cells.

## DISCUSSION

The generation of cardiomyocytes from iPSCs *in vitro* was possible by controlling the gene regulatory network during stepwise fate transitions (Alexander and Bruneau, 2010). However, there are similar characteristics between artificially-induced pluripotency and oncogenic transformation, and the possibility of developing malignancy in iPSC-derived cells is still present at later time points (Nedelcu and Caulin, 2016; Romano et al., 2014). In this study, our single-cell transcriptome sequencing and bulk RNA sequencing revealed that the dynamic expression of oncogenes and TSGs was present in various stages of human iPSC-derived cells, including the Yamanaka factor and p53 genes, which are activated or silenced in a certain part of human iPSC-derived cardiomyocytes.

To date, very few oncogenes or TSGs have been dissected in human iPSCs and iPSC-derived cells at the single-cell level, and little information is known about the predominant mechanisms linking cancer-related gene and cardiac differentiation *in vitro*. Oncogenes refer to the sequences of DNA whose alterations may cause gain-of-function effects and their transcription products may be able to induce cancer (Osborne et al., 2004). TSGs are known as anti-oncogenes, and they often function as cellular gatekeepers and checkpoints for normal cell growth and division (Plank and Henske, 2000). Like other genes in different kinds of cells, their transcriptional products could be proteins and noncoding RNAs, and more evidence has revealed that some microRNAs and long noncoding RNAs are carcinogenic factors (Liang et al., 2015). There are some public data resources for oncogenes and TSGs, such as the UniProtKB database, OncomiRDB, ONGene, and TSGene databases, among which, ONGene, and TSGene are literature-based databases (Liu et al., 2017a; Wang et al., 2014; Zhao et al., 2016). These databases are valuable for the assessment of the roles of cancer-related genes in human carcinogenesis. By searching these databases, we can find a portion of the cancer-related genes expressed during our cardiac differentiation experiment, including the Yamanaka factor and p53 genes. In the literatures, the Yamanaka factor and p53 genes have been recognized as promising molecules that play important roles in cell growth, differentiation and apoptosis (Curry et al., 2015; Gong et al., 2016). Yamanaka factors include four factors: Oct4, Sox2, Klf4, c-Myc; and these factors have been discovered as the core regulators in somatic cell reprogramming in recent years (Takahashi and Yamanaka, 2006). In fact, the Yamanaka factors have been reported to be frequently upregulated in cancers. Moreover, their expression levels are often associated with the increased metastatic potential of cancer cells and a poor clinical prognosis (Wasik et al., 2014). In contrast, p53 is well recognized as a tumor suppressor, which plays roles in converting different kinds of cellular upstream signals into downstream responses (Koyama et al., 2017; Wu, 2004). Previous studies have revealed that manipulating the p53 expression level may contribute to higher reprogramming efficiency in human iPSC generation and cardiac differentiation (Kawamura et al., 2009; Wang et al., 2018). However, p53 has been found to be mutated or lost in approximately half of all human cancers, and it is also a key factor in cancer progression and metastasis (Powell et al., 2014). Our present study revealed that some well-known oncogenes, such as Yamanaka factor genes were expressed at a relatively low levels at the whole cell population level, but their expression signatures vary greatly between cells, even when the same cell line and culturing conditions are used. And although there was no direct evidence to support downregulation of *TP53* in the cardiomyocytes compared to other cell states, we revealed *TP53* silence in a subset of *TNNT2*+ and *MYC*+ cardiomyocytes. This may imply that the cardiomyocytes generated from human iPSCs may be heterogeneous, and their functions and safety may need to be assessed at the single-cell level.

In the process of oncogenesis, proto-oncogene activation and TSG defection may not be isolated events (Daya-Grosjean and Sarasin, 2005). In the present study, single-cell sequencing enabled us to simultaneously identify cancer genes in each single cell. Interestingly, by focusing on the well-known cancer genes, we discovered that some of our human iPSC-derived *TNNT2*+ cardiomyocytes were *MYC*+ and *TP53*-, including that although proto-oncogene activation and TSG defection are not commonly found in the majority of cells, they may be present in some human iPSC-derived cardiomyocytes. In the majority of *MYC*+ cardiomyocytes, *MYC* is expressed at a very high level; however, over half of *MYC*+ cardiomyocytes are *TP53*-, and the rest of them are expressed *TP53* at very low levels. Although Yamanaka factors may trigger the activation of p53, our data indicate that *MYC*+ cardiomyocytes tend to silence p53 rather than upregulate the expression of the stress response genes (Wasik et al., 2014). c-Myc overexpression in conjunction with p53 inactivation in some human iPSC-derived cardiac cells may further accelerate the spontaneous development of genomic instability and may increase the cell carcinogenesis risk (Abba et al., 2004). Furthermore, this molecular event, such as *MYC* amplification and *TP53* silence, is not unique, and we could find a small population (6.85%) of human iPSC-derived cardiomyocytes with *MYC*+ and *TP53*- in the other single-cell RNA sequencing dataset (Friedman et al., 2018). Therefore, the oncogene and TSGs involved in the gene regulatory network of cardiac differentiation are worthy of attention.

Our study provides new insights into the dynamic expression of oncogenes and TSGs in the generation of cardiomyocytes from human iPSCs at single-cell resolution, which are difficult to be detected by bulk transcriptional sequencing. And the co-occurrence of *MYC* overexpression and *TP53* silencing in the cardiomyocytes may be a clue to understand the carcinogenic risk of human iPSC-derived cells.

## MATERIALS AND METHODS

### Cell culture and sample preparation

To explore cell reprogramming at the single-cell level, we adopted a well-established protocol to generate human iPSC-derived cardiomyocytes by using small molecule-based methods (Jiang et al., 2018; Lan et al., 2013). In detail, two commercially available human iPSC lines (CA4024106, cell line 1 M-iPS; CA4027106, cell line 2 F-iPS) (Cellapy, Beijing, China). These cell lines were previously generated from health males and females using Cytotune-iPS 2.0 KOSM transgenes without inhibiting p53 expression, which were recently authenticated and negative for contamination; and the two cell lines were used for cardiac differentiation by a chemically defined cardiac differentiation kit (CA2004500) (Cellapy, Beijing, China) (Jiang et al., 2018). Briefly, the two human iPSC lines were resuscitated and subjected to pluripotency and karyotype confirmation before cardiac differentiation. Then, the two cell lines were kept and cultured in chemically defined PSCeasy medium. When reaching a confluence of 80∼90% (day 0, 0 h), these cells were cultured in Induction Medium I at 37°C with 5% CO2 for 48 h. After that the medium was changed to Induction Medium II and was cultured at 37°C with 5% CO2 for another 48 h; then, the medium was changed to Induction Medium III, and fresh Induction Medium III was supplied afterwards every other day. Considering the principle of the cardiac differentiation kit and the known marker gene expression in the literature, the cell culture samples were collected at four different time points in the process of cardiac differentiation, including at the time points of day 0 (0 h), day 2 (48 h), day 4 (98 h), and day 10 (240 h). For cell suspension preparation, the cell samples were harvested from adherent cultures by accutase and EDTA method. Each cell sample collected from different time points was subjected to single-cell RNA sequencing, bulk RNA sequencing and marker gene expression detection using immunofluorescence technology.

### Single-cell RNA sequencing library preparation and sequencing

For each time point of cell collections in the cardiac differentiation process, four differentiations, including two differentiations collected from cell line 1 and another two differentiations collected from cell line 2, were harvested for single-cell RNA sequencing. The cell samples were suspended in phosphate-buffered saline (PBS) at a density of 700 cells μl^−1^; then the cells were used for single-cell RNA sequencing library preparation by Chromium Single Cell 3′ Reagent Kit (version 2) and Chromium Controller (10X Genomics, CA, USA) according to the manufacturer's instructions. In brief, the cell samples at each time point were suspended at 1×106 cells, and the cell samples with more than 90% of the cells viable as determined by Trypan Blue staining were chosen for further analysis. For per reaction, approximately 10,000 living cells, including 5000 cells from cell line 1 and 5000 from cell line 2, were loaded for the generation of gel bead-in-emulsion (GEM). In total, four reactions were performed for the four groups of samples (iPS-0, iPS-1, iPS-2, and iPS-3) collected from day 0, day 2, day 4, and day 10. The released RNA from each captured cell in individual GEM was barcoded by reverse transcription. The barcoded cDNAs in different GEM were pooled and cleaned. The cDNAs were further amplified and sequenced by PE150 on Illumina NovaSeq platform (Illumina Inc., San Diego, CA, USA).

### Single-cell data preprocess, alignments and gene expression signature analysis

The raw sequencing data were converted to short reads. The FASTQ files with short reads were preprocessed using Trimmomatic software, including removing the low-quality reads, trailing or N bases, adapters, reads less than 26 bp and reads that could not form paired reads. The remaining clean reads were used for further analysis. The basic statistics of the reads were performed using FastQC software. Next, we used a set of analysis pipelines in the Cell Ranger Single-Cell Software Suite for data merging, barcode processing, gene counting and alignments (Zheng et al., 2017). Briefly, the splicing-aware alignment of the sequencing reads to the human genome (GRCh38) was performed via STAR. Then, the reads aligned confidently to the human genome were bucketed into exonic, intronic, and intergenic by the transcript annotation GTF as follows: (1) exonic, at least 50% of bases intersected an exon; (2) intronic and non-exonic base; or (3) intergenic otherwise. For the reads aligning to one single exonic locus and aligning to multiple loci at the same time, the exonic locus was chosen as the representative locus, and the read with MAPQ 255 was considered mapping to the exonic locus confidently. The exonic reads were further annotated, and those that mapped to transcriptome confidently were further used for unique molecular identifier (UMI) counting. For the cell counting, the expected number of recovered cells (N) was taken as input in Cell Ranger analysis, and its internal normalize method was adopted for normalizing the data (Bach et al., 2017; Cole et al., 2017; Tsang et al., 2017). The maximum total UMI counts were robustly estimated by m, which was taken as the 99th percentile of the top N barcodes by the total UMI counts. All barcodes with total UMI counts greater than m/10 were identified as cells. To display the most important cell features, the gene expression matrix was reduced by Cell Ranger via Principal Components Analysis (PCA), and the dimensionality of the cell dataset from (i.e. cells x genes) was changed to (cells×M), where M was the number of principal components; the PCA-reduced data was then clustered by Cell Ranger via k-means algorithm with default parameters. The cell subpopulations in cardiac differentiation were visualized in two-dimensional images by t-Stochastic Neighbor Embedding (t-SNE) and the cell types were identified by feature plot using Loupe Cell Browser (10×Genomics). The number of the clusters (k value) was chosen by the sum of the squared errors and silhouette coefficient; differentially expressed genes in the cells each cluster were detected with a likelihood-ratio test and an adjusted *P*-value of 0.05 was set as statistical significance threshold (Liu et al., 2019); and the marker genes that were specific to each cluster were identified and displayed by the Loupe Cell Browser. The pseudotemporal ordering of single cells in the cell subpopulations was performed using the package Monocle (Trapnell et al., 2014) (https://cole-trapnell-lab.github.io/monocle-release/docs/), and the genes in each cluster that are unusually variable across all of the cells in the whole reprograming were screened and chosen by clusterCells (min_expr=0.5); the chosen genes were used to order the cells by orderCellsvia via Monocle, and each cluster of the cells in the cell differentiation trajectory was displayed. For Gene Ontology (GO) enrichment analysis, the ClusterProfiler R package was used, and *P*<0.05 was considered significantly enriched (Yu et al., 2012).

### Bulk RNA sequencing

Three differentiations of each cell line at each time point were collected and pool for cell suspension preparation, and total eight groups of samples were subjected to bulk RNA sequencing. The bulk RNA sequencing was performed according to our previous studies with minor modifications (Ou et al., 2014; Tan et al., 2013). The total RNA of the cells was extracted by TRIzol (Invitrogen), and 3 μg RNA was taken for each transcriptome library preparation using NEBNext^®^ UltraTM RNA Library Prep Kit for Illumina^®^ (NEB, USA) according to the manufacturer's instructions. Briefly, the cDNA was synthesized from the purified mRNA and adaptors were added. After library fragmentation and purification, the adaptor-ligated cDNA was amplified using Universal PCR Primers, Index (X) Primer and Phusion High-Fidelity DNA Polymerase. The PCR products were further purified, and library quality control was performed using the Agilent Bioanalyzer 2100 system. After cluster generation, each library was sequenced using the Illumina HiSeq platform, and paired-end reads were generated. Clean reads were obtained from the raw data by removing low quality reads and reads containing adapters and ploy-N. The clean reads were mapped to the reference genome by Hisat2 v2.0.5, and expression gene analysis was performed using the DESeq2 R package (1.16.1).

### Immunofluorescence

For the confirmation of marker gene expression, the two cell lines were seeded and grown on coverslips with the same condition as the cells using for RNA sequencing. The coverslips with cells were collected, fixed with 4% paraformaldehyde for 15 min, and washed with PBS at room temperature three times. The samples were then treated with Triton 0.3% X-100 (Sigma-Aldrich) for 20 min, and incubated with normal blocking buffer and washed with PBS three times; then the samples were incubated in BSA (1%) (Sigma-Aldrich) with primary antibodies overnight at 4°C. After washing three times with PBS, the samples were incubated for 60 min at 37°C in BSA (1%) (Sigma-Aldrich) with secondary antibodies. The primary and secondary antibodies were as follows: anti-POU5F1 (Santa Cruz Biotechnology, SC-9081, used at a 1:100 dilution) and goat anti-rabbit IgG (Life Technologies, A11008, used at a 1:200 dilution); anti-NANOG (Abcam, Ab21624, used at a 1:100 dilution) and goat anti-rabbit IgG (Life Technologies, A11008, used at 1:200 dilution); anti-PODXL (Santa Cruz Biotechnology, SC-21705, used at a 1:100 dilution) and goat anti-mouse IgG (Life Technologies, A11005, used at a 1:200 dilution); anti-SSEA4 (Santa Cruz Biotechnology, SC-21704, used at a 1:100 dilution) and goat anti-mouse IgG (Life Technologies, A11005, used at a 1:200 dilution); anti-Brachyury (Abcam, Ab20680, used at a 1:100 dilution) and goat anti-rabbit IgG (Life, A11001, used at a 1:200 dilution); anti-GATA4 (Millipore, Ab4132, used at a 1:100 dilution) and goat anti-rabbit IgG (Life Technologies, A11008, used at a 1:200 dilution); anti-TNNT2 (Abcam, Ab8295, used at a 1:200 dilution) and goat anti-mouse IgG (Life Technologies, A11012, used at a 1:200 dilution); anti-ACTN2 (Sigma-Aldrich, A7811, used at 1:200 dilution) and goat anti-mouse IgG (Life Technologies, A11012, used at a 1:200 dilution). The nuclei of the cells were counterstained with DAPI (Vector Laboratories, Burlingame, USA), and the images were taken by a fluorescence microscope (BX51) (Olympus, Tokyo, Japan).

### Statistical analysis

For the data of the single-cell sequencing, four groups of samples collected at different time points in the cardiac differentiation were used, and their gene-cell barcode matrix was concatenated; and the single and pooled matrixes was used for PCA, tSNE and clustering (k-means). The primary data of single cell sequencing and bulk RNA sequencing was collected and organized by Microsoft Excel (Microsoft office professional plus 2010) and Notepad++ (Version 7.6.4). And all of the statistical analyses without specified in this study were performed by Graphpad Prism (Version 8.0). For the potential regulation network of the expressed cluster specific marker genes and cancer gene analysis, we calculated the correlation scores and corresponding statistical *P*-values for all the marker genes and cancer genes using their average UMI in different cell clusters by the R language package (version 3.14.0). For all the resulting *P*-values for the correlation test, we further checked the false discovery rate (FDR) to correct the statistical significance of multiple testing. To explore the potential molecular connection for the interested 24 oncogenes and tumor suppressors, we utilized the STRING to build the functional protein association network (reference: https://pubmed.ncbi.nlm.nih.gov/30476243/). Essentially, the 24 genes were uploaded to STRING online server and choose the Homo Sapiens as the background network. Then the STRING predicted all the interactions based on their neighborhood on chromosome, gene fusion data, phylogenetic co-occurrence, homology interaction, gene co-expression data, experimentally determined interaction, annotated protein-protein interaction database, and the text mining for those genes. The final gene coexpression network was visualized by Cytoscape (Smoot et al., 2011).

## Supplementary Material

Supplementary information
