# Supplementary material for: Single-cell sequencing reveals the potential oncogenic expression atlas of human iPSC-derived cardiomyocytes
Source: Biol Open. 2021 Feb 15;10(2):bio053348. doi: 10.1242/bio.053348 (PMC7903994; doi:10.1242/bio.053348)
Supplement: Supplementary information [file biolopen-10-053348-s1.pdf]

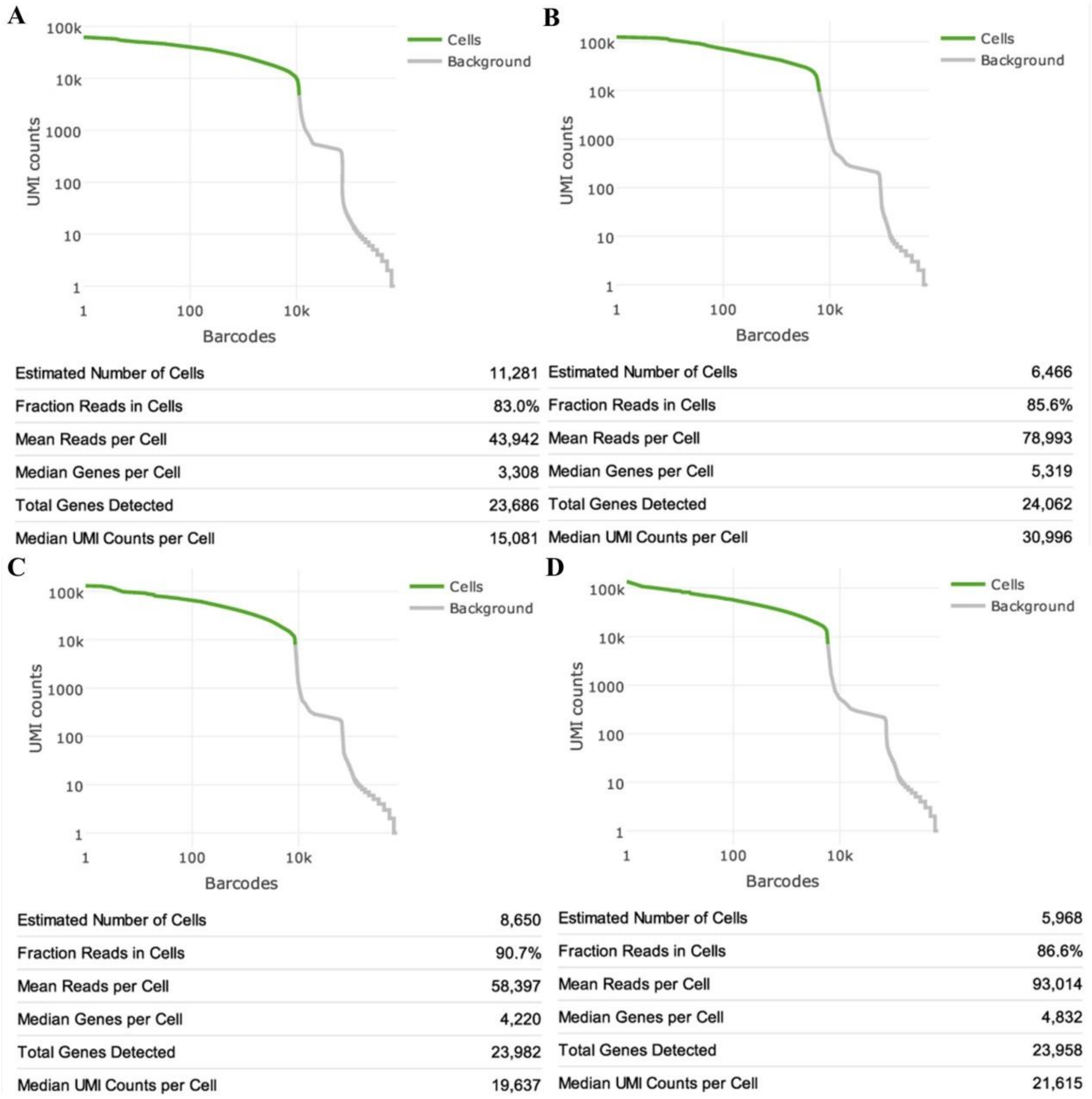

A

Estimated Number of Cells	11,281
Fraction Reads in Cells	83.0%
Mean Reads per Cell	43,942
Median Genes per Cell	3,308
Total Genes Detected	23,686
Median UMI Counts per Cell	15,081

B

Estimated Number of Cells	6,466
Fraction Reads in Cells	85.6%
Mean Reads per Cell	78,993
Median Genes per Cell	5,319
Total Genes Detected	24,062
Median UMI Counts per Cell	30,996

C

Estimated Number of Cells	8,650
Fraction Reads in Cells	90.7%
Mean Reads per Cell	58,397
Median Genes per Cell	4,220
Total Genes Detected	23,982
Median UMI Counts per Cell	19,637

D

Estimated Number of Cells	5,968
Fraction Reads in Cells	86.6%
Mean Reads per Cell	93,014
Median Genes per Cell	4,832
Total Genes Detected	23,958
Median UMI Counts per Cell	21,615

Figure S1. Summary of the single-cell sequencing output data of different sample groups. (A) the sample collected on day 0; (B) the sample collected on day 2; (C) the samples collected on day 4; (D) the samples collected on day 10.

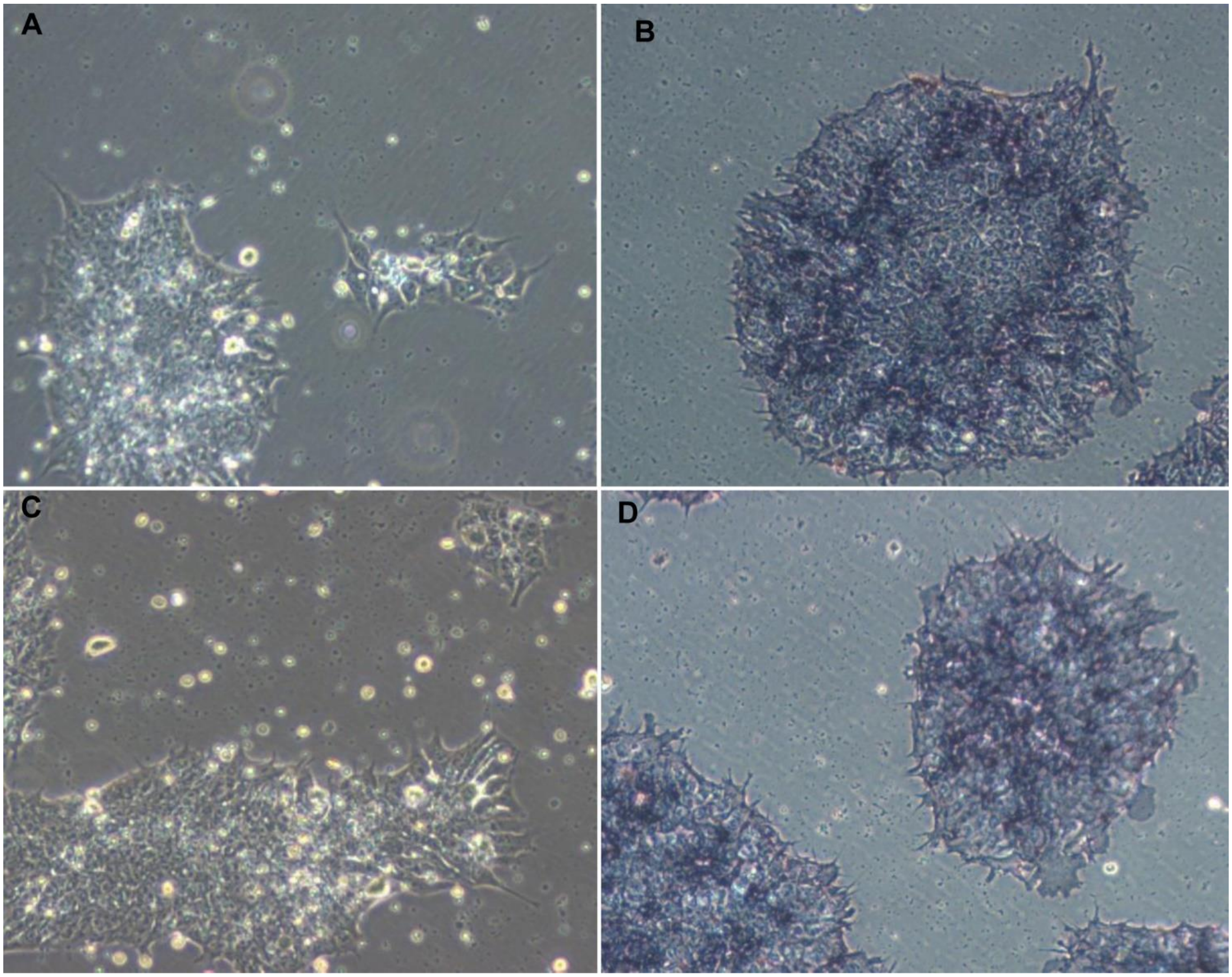

Figure S2. Cell morphology and alkaline phosphatase staining of the two iPSC lines. (A) Cell morphology of cell line 1 in bright fields of microscope; (B) cell line 1 showed strong expression of alkaline phosphatase; (C) Cell morphology of cell line 2 in bright fields of microscope; (D) cell line 2 showed strong expression of alkaline phosphatase.

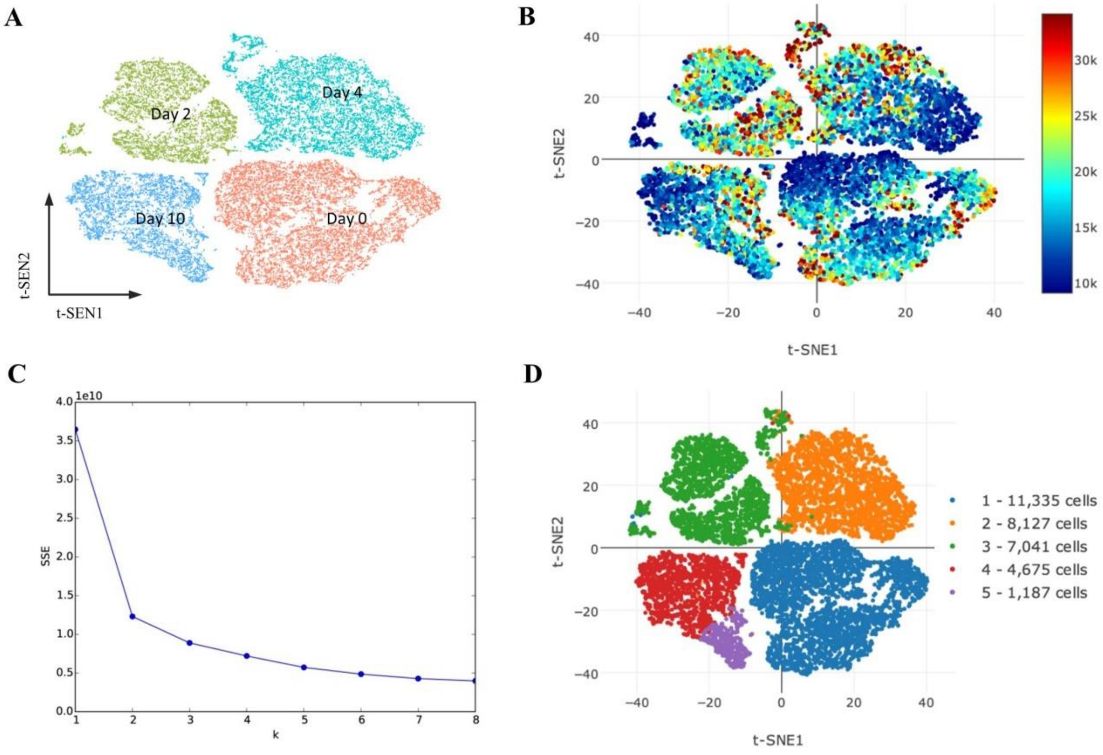

Figure S3. Subpopulations of the cell in cardiac differentiation. (A) t-SNE displaying the four clusters of cells collected at the four differentiation time points in cardiac differentiation; (B) t-SNE displaying the cells colored by UMI counts; (C) optimal partitioning of human iPSC-derived cells using k-means with k=5 clusters via the sum of the squared errors as determined above; (D) t-SNE displaying the five clusters of cells partitioning k-means (k=5).

Table S1: The stage-specific genes emerged in the cardiac differentiation.

Click here to Download Table S1

Biology Open • Supplementary information
